# Potential Health Impacts, Treatments, and Countermeasures of Martian Dust on Future Human Space Exploration

**DOI:** 10.1029/2024GH001213

**Published:** 2025-02-12

**Authors:** Justin L. Wang, Jeremy J. Rosenbaum, Ajay N. Prasad, Robert R. Raad, Esther J. Putman, Andrea D. Harrington, Haig Aintablian, Brian M. Hynek

**Affiliations:** ^1^ Keck School of Medicine University of Southern California Los Angeles CA USA; ^2^ Ann and HJ Smead Department of Aerospace Engineering University of Colorado Boulder CO USA; ^3^ Astromaterials Acquisition and Curation Office NASA Johnson Space Center Houston TX USA; ^4^ Space Medicine University of California Los Angeles Los Angeles CA USA; ^5^ Laboratory for Atmospheric and Space Physics University of Colorado Boulder CO USA; ^6^ Department of Geological Sciences University of Colorado Boulder CO USA

**Keywords:** medical geology, artemis, pulmonology, silicosis, astronaut, Mars

## Abstract

The challenges of human space exploration produce some of humanity's greatest technological and scientific advances, not excluding innovations in medicine. The microgravity environment causes a host of physiological changes, and exposure to dust on the Moon caused considerable pulmonary distress to astronauts during the Apollo missions. As the National Aeronautics and Space Administration and other organizations prepare for long‐duration exploration missions to Mars, the hazards and consequences of the Martian surface need to be accounted for. This review investigates how substances analogous to hazardous components of Martian dust have caused disease in people on Earth. Because of its small grain size, dust on Mars is more likely to cause lung irritation, absorb into the bloodstream, and lead to diseases in astronauts. Toxic components of martian dust include perchlorates, silica, nanophase iron oxides, and gypsum in addition to trace amounts of toxic metals whose abundances are debated: chromium, beryllium, arsenic, and cadmium. Predicted effects of dust exposure ranges from asymptomatic to life‐threatening, with many substances being carcinogenic and most damage impacting the pulmonary system. The longer transit time for astronauts to return home makes the operations of performing emergency medical treatment more difficult and increases both the likelihood and consequences of developing chronic disease. Exposure mitigation needs to be prioritized; however, supplements may be taken to prevent disease from breakthrough exposures, and treatment regimens could lessen morbidity and mortality. Treatments and equipment need to be carefully considered and transported with the astronauts to be prepared for all possible scenarios.

## Key Points

- From microgravity to harmful radiation, human space exploration causes physiological changes, and a journey to Mars introduces novel issues
- The small size, oxidative properties, and chemical composition of Martian dust may cause disease in astronauts, particularly in the lungs
- Dust filtration, cabin cleaning, and disease treatments need to be considered to ensure astronaut health and mission success on Mars

## 1 Introduction

There are many challenges to achieving the National Aeronautics and Space Administration's (NASA) goal of a human mission to Mars. Many technological issues of a manned Mars mission have been explored through the Mars Exploration Program and will continue to be addressed by early Artemis missions to the Moon, but complex challenges still remain in the field of human health protection. These challenges include physiological adaptations during long‐duration spaceflight and microgravity exposure, which have been studied extensively on missions to the International Space Station (ISS).

In brief, the lack of normal bodily stresses from gravity leads to a self‐limiting muscle loss and a non‐self‐limiting bone loss. This bone loss can lead to increased urinary calcium, which can precipitate kidney stones. Additionally, the microgravity‐induced fluid shift leads to a ∼11% total blood volume reduction, cardiac atrophy, and arrhythmias (Buckey, 2006). This fluid shift also causes changes in the vestibular system such as reduced balance and space motion sickness (Buckey, 2006). Immunological changes have also been assessed, including reactivation of latent viruses and impairment of acquired immunity (Akiyama et al., 2020). Current spacesuit designs require low operating pressures to maintain mobility, which introduces the risk of decompression sickness. Another hazard of particular importance is damaging ionizing radiation coming from the sun in the form of solar energetic particles and from galactic cosmic radiation. The Earth's magnetosphere shields many of these damaging particles for astronauts in Low Earth Orbit on the ISS and, to a degree, on the lunar surface when the Moon lies within Earth's magnetotail. A human mission to Mars, however, will not have the same protection, and potential health issues include cataract development, cancer, pulmonary fibrosis, and damage to the central nervous system (Buckey, 2006; Christofidou‐Solomidou et al., 2015). In addition to higher and protracted levels of radiation exposure, astronauts venturing to Mars will face novel health risks that come with exploring a new planetary surface.

**Figure 1:**
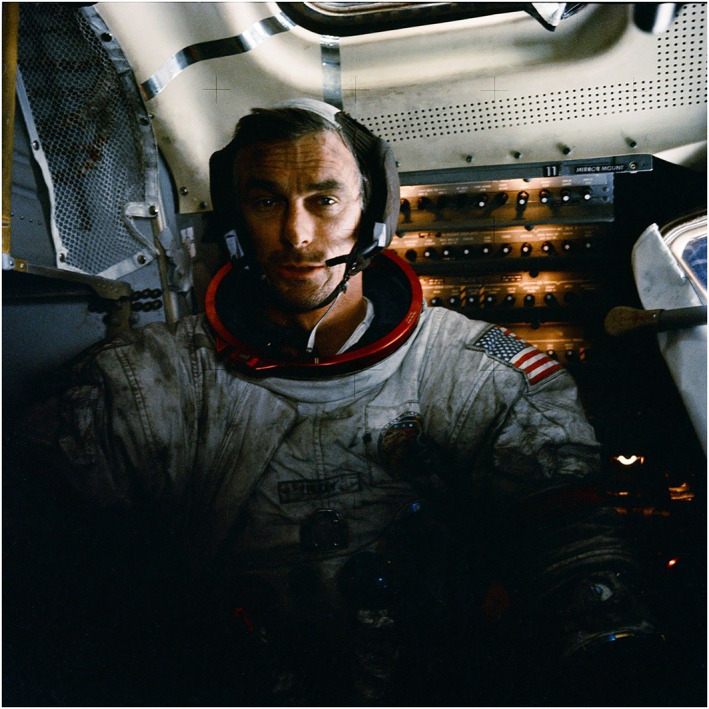
Lunar dust covering astronaut Gene Cernan's spacesuit during Apollo 17 (NASA, 2017).

Lunar dust and its risk to astronaut health has been extensively studied, and Pohlen et al. (2022) provided a review of this subject. Due to a lack of erosion on the Moon, dust was abrasive and noxious to the Apollo astronauts (Figure 1). Reactions with galactic and solar radiation lead to the development of nanophase iron, which makes the dust magnetic, electrostatic, and toxic due to the formation of reactive oxygen species (ROS) when in contact with human tissue. Furthermore, this electrostatic charge made dust adhere easily to astronauts' spacesuits, which then introduced dust into the lunar habitat. The most reported symptoms were cough, throat irritation, and erythematous, watery eyes accompanied by decreased vision. Samples returned from the Moon from Apollo have been studied in labs to assess astronaut risk, and in 2014, the Lunar Airborne Dust Toxicity Advisory Group (LADTAG) determined a permissible exposure limit of 0.3 mg/m^3^ for a 6‐month lunar mission with eight hours of lunar dust exposure for five days per week (James et al., 2014). While only short‐term symptoms developed in the Apollo astronauts, results from multiple investigations suggest that prolonged exposure may cause chronic effects. Pohlen et al. (2022) discuss potential countermeasures for lunar dust toxicity, such as dust removal technologies, HEPA filters, air quality monitors, and suitports, which are also all relevant to limiting dust exposure on Mars.

**Figure 2:**
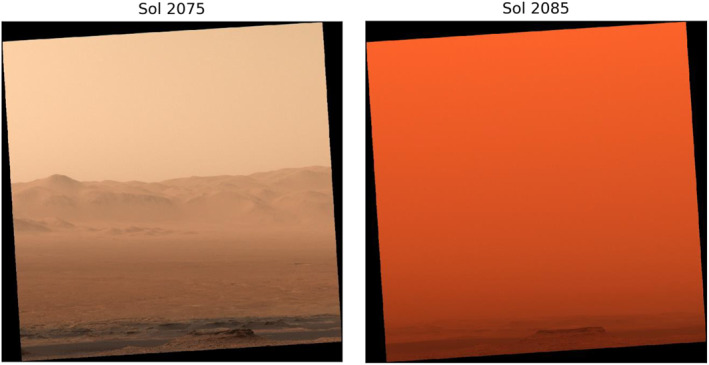
Curiosity's view of Gale Crater during the 2018 global dust storm on Mars showing a decrease in visibility from 8 June 2018 (Sol 2075) to 18 June 2018 (Sol 2085) (NASA/JPL‐Caltech/York University, 2019).

While dust on Mars is not as sharp and abrasive as lunar dust due to increased erosion on the planet's surface, it shares many properties with lunar dust and still poses a risk to astronauts. Estimates have shown that dust on Mars is electrostatic, magnetic, abrasive, highly oxidative, chemically reactive, irregularly shaped with rounded edges, and has a diameter from ∼2 μm up to ∼8 µm during dust storms (Figure 2) with an average diameter of ∼3 μm (Lemmon et al., 2019; Ming & Morris, 2017; National Research Council, 2002).

First‐line defenses to martian dust exposure include physical barriers from spacesuits and human habitats. Several technologies have been developed to limit contaminant exposure, including habitat air filters, electrostatic repulsion devices, and self‐cleaning functions on spacesuits (Afshar‐Mohajer et al., 2015; Manyapu et al., 2019). Nevertheless, the dust's electrostatic, magnetic, and fine‐grained qualities make contamination inevitable. This will likely resemble what occurred during the Apollo missions when electrostatic dust adhered on astronauts' spacesuits after extravehicular activities (EVAs) and was then carried into the living quarters through the airlock and dispersed within the module (Pohlen et al., 2022).

No samples of martian dust have been analyzed in Earth laboratories, and while remote sensing and modeling have revealed many properties of the martian atmosphere, such as a likely bimodal particle size distribution (Esposito et al., 2011; Määttänen et al., 2024), its specific composition, shape, size, and uniformity across the planet's surface remain uncertain for now. The Mars Sample Return Campaign was kicked off with the launch of the Perseverance Rover in 2020. Although the current campaign architecture is currently under evaluation at the time of this manuscript publication, it is anticipated that samples may be returned to Earth as early as the mid‐to late 2030's. Although there are numerous science goals associated with the study of martian samples, preparing for human exploration of Mars is a mission priority (Beaty et al., 2019). Several studies from rover experiments and orbiter data, however, have identified potentially toxic minerals and chemicals in martian dust (National Research Council, 2002). The study of pristine martian materials on Earth, particularly regolith and airborne dust, could help clarify potential hazards and mitigate risks to astronauts. This review investigates how these geological toxins have caused health issues for humans on Earth, the treatments used to help with disease management, and potential countermeasures that can be used to promote a safe, manned exploration of Mars.

## 2 Geological Hazards, Pathophysiology of Associated Disease, and Treatments

Exposure to martian dust may come from dermal exposure, ocular contact, ingestion, or inhalation through oral and nasal cavities. The severity of pulmonary disease makes inhalation of dust a primary concern for crewmember health. As described previously, the average diameter of martian dust is ∼3 μm. The majority of this dust will likely penetrate the physical innate immune defenses of the respiratory tract as mucus in the lungs is not able to expel dust particles that have a diameter of less than 5 μm (Davila et al., 2013). Thus, dust on Mars is more likely to cause lung irritation, absorb into the bloodstream, and lead to diseases in astronauts.

As astronauts are already medically predisposed to certain diseases from spaceflight and enhanced radiation exposure, geological health hazards on Mars must be considered in the overall context of the altered human body during space travel. Mars' unique geological landscape and subsequent dust composition introduce a collection of challenging health risks, which are introduced in this section and summarized in Table 1.

**Table 1:** A Summary of the Geological Health Hazards on Mars Accompanied With Abundance, Associated Disease, and Potential Treatments or Countermeasures

Hazard	Abundance	Health effects	Treatment or countermeasure
Small aerosol dust particles	Uniform on Mars; Diameters range from ∼2 to ∼8 μm with an average of ∼3 μm	Dust with diameter <5 μm cannot be expelled by lung mucus and are absorbed into the bloodstream	Filtration; Dust removal
Perchlorates	Global distribution	Thyroid impacts causing aplastic anemia	Potassium iodide oral supplement for disease prevention
Silica	Global distribution	Restrictive lung disease (Silicosis); renal effects; immunological effects	No cure; Supportive treatment for lung disease (oxygen, bronchodilators, cough medicine)
Basalt and Pyrite	Global distribution (Basalt); Martian meteorite detection (Pyrite)	Lung disease	No cure; Supportive treatment for lung disease (oxygen, bronchodilators, cough medicine)
Nanophase iron oxides	Global distribution	Lung disease; infection susceptibility	No cure; Supportive treatment for lung disease (oxygen, bronchodilators, cough medicine)
Gypsum	Olympia Undae Sand Sea; Juventae Chasma; Meridiani Planum; Columbia Hills	Gastrointestinal blockage (consumption); fibrotic lung disease (inhalation)	No cure; Supportive treatment for lung disease (oxygen, bronchodilators, cough medicine)
Chromium (VI)	Pathfinder discovered Chromium in soil; Global abundance of Cr(VI) is undetermined	Fibrotic lung disease; Hemorrhagic gastroenteritis; Ulceration of the nasal septum	Vitamin C oral supplement for disease prevention; Supportive treatment for lung disease (oxygen, bronchodilators, cough medicine)
Beryllium	Trace amounts from Martian meteorites	Restrictive lung disease (Berylliosis)	No cure; Supportive treatment for lung disease (oxygen, bronchodilators, cough medicine)
Cadmium	Trace amounts from Martian meteorites	Interstitial pneumonia; Kidney disease; Osteoporosis & osteomalacia	Gastric lavage for acute exposure; No cure for chronic exposure
Arsenic	Trace amounts from Martian meteorites	Neuropathy; Carcinogenic	Gastric lavage or activated carbon for acute exposure; No cure for chronic exposure

Mars' crust is mainly composed of igneous mafic to ultramafic rocks that are typically classified as basalts. The elemental composition of the crust is similar to that of the Moon, with Si > Mg > Fe. Notably, Mars has higher amounts of Fe and S (and other volatiles) relative to the Moon (Table 2) (*e.g.*, Taylor, 2013). Unlike the Moon, Mars experienced extended (millions of years) of wet conditions at the surface (*e.g.*, Hoke et al., 2011) that led to pervasive chemical weathering of the basaltic crust and alteration and concentration of secondary minerals. The original rocks were transformed into a variety of weathered products, including abundant sulfate minerals, clay minerals (phyllosilicates), chlorides/chlorates, and occasional carbonates (*e.g.*, Carter et al., 2013). These have been observed up close by landed spacecraft and it is clear that fluids have led to strong enhancements of chloride and sulfate minerals in the near surface (e.g., Hecht et al., 2009; Hynek et al., 2019). Some of these aqueously altered products, which have no lunar counterpart, as well as the bulk primary minerals/elements in the crust, can be significant human health hazards.

**Table 2:** Martian Crust Bulk Compositions From Taylor (2013)

Bulk silicate oxide composition	Concentration	Bulk silicate oxide composition	Concentration	Bulk silicate oxide composition	Concentration	Bulk silicate oxide composition	Concentration	Major element composition	Concentration
Li (ppm)	3.0	Co (ppm)	71	Ag (ppb)	4.2	Ho (ppb)	106	SiO_2_ (wt %)	43.7
Be (ppb)	47.7	Ni (ppm)	330	Cd (ppb)	9.6	Er (ppb)	306	TiO_2_ (wt %)	0.14
F (ppm)	21	Cu (ppm)	2.0	In (ppb)	6.9	Tm (ppb)	44.8	Al_2_O (wt %)	3.04
Na (%)	0.40	Zn (ppm)	18.9	Sn (ppb)	38.5	Yb (ppb)	308	Cr_2_O_3_ (wt %)	0.73
Mg (%)	18.5	Ga (ppm)	6.6	I (ppb)	36	Lu (ppb)	44.8	FeO (wt %)	18.1
Al (%)	1.64	As (ppb)	86	Cs (ppb)	95	Hf (ppb)	217	MnO (wt %)	0.44
Si (%)	20.5	Se (ppb)	85	Ba (ppm)	4.37	Ta (ppb)	27.2	MgO (wt %)	30.5
P (ppm)	675	Br (ppb)	191	La (ppb)	439	W (ppb)	74	CaO (wt %)	2.43
Cl (ppm)	32	Rb (ppm)	1.30	Ce (ppb)	1,170	Re (ppb)	0.88	Na_2_O (wt %)	0.53
K (ppm)	309	–	–	Pr (ppb)	176	Os (ppb)	2.0	K_2_O (wt %)	0.04
Ca (%)	1.74	Sr (ppm)	14.6	Nd (ppb)	864	Ir (ppb)	2.0	P_2_O_3_ (wt %)	0.15
Sc (ppm)	11.0	Y (ppm)	2.89	Sm (ppb)	274	Pt (ppb)	3.1		
Ti (ppm)	832	Zr (ppm)	7.49	Eu (ppb)	103	Tl (ppb)	1.28		
Cr (ppm)	4,990	Nb (ppb)	501	Gd (ppb)	374	Bi (ppb)	0.60		
Mn (%)	0.34	Ru (ppb)	2.6	Tb (ppb)	67.3	Th (ppb)	58		
Fe (%)	71	Pd (ppb)	2.4	Dy (ppb)	450	U (ppb)	16		

The highly oxidative anion perchlorate (ClO_4_^−^) was detected in abundance at the landing site of the 2007 Phoenix lander (Hecht et al., 2009) as well as at the 2011 Curiosity landing area (Clark et al., 2021). It is now clear that perchlorates occur globally on Mars (Clark & Kounaves, 2016). Inhaled perchlorate presents a challenge for astronauts as they traverse Mars for its impacts on hormonal regulation. The major side effect associated with the prevalence of perchlorate in martian dust (0.5%–1% of soil) (Davila et al., 2013) is aplastic anemia from thyroidal impacts (Hobson, 1961). The current safe exposure limit is about 0.0007 mg/kg body weight/day ‐ which translates to a few milligrams of martian dust easily surpassing the recommended dose (Agency for Toxic Substances and Disease Registry, 2008). The highly oxidized chlorine is hypothesized to block thyroid function by acting as a competitive inhibitor for the sodium‐iodide symporter (NIS) found on the basolateral membrane of thyroid cells (Leung et al., 2010). By decreasing the efficacy of NIS, iodide availability decreases, which can cause growth issues as iodine is a major building block for thyroid hormones. In one case study, a patient was given 800 mg/day of perchlorate for 14 weeks and a subsequent decrease to 600 mg/day for another 18.5 weeks. This amount of perchlorate caused severe aplastic anemia, which resulted in thrombocytopenia, increased bleeding time, and infection susceptibility. The patient died from a pulmonary infection following the 18.5 weeks despite corticosteroid and antibiotic therapy (Hobson, 1961).

There are a few proposed solutions to address sources of concern relating to perchlorates. To begin with, iodine intake can be increased when there is a known, continuous exposure to inhaled martian dust (Lewandowski et al., 2015). Although there can be side effects to iodine overuse, this can be carefully monitored through blood draws and a nutritional balance. The binding of perchlorates to NIS, although harmful, is reversible and not found to cause chronic issues. Notably, the selective reduction of perchlorates can be achieved using anaerobic enzymatic digestion. Through a biogeochemical redox reaction, perchlorate can be chemically modified to chlorite, and subsequently chlorine and oxygen through perchlorate reductase and chlorite dismutase, respectively (Coates & Achenbach, 2006). Although in need of specific biochemical testing and further technological development, this process could potentially reduce the concentration of perchlorate in an active air filtration system for astronauts. As with the Phoenix lander results, perchlorate can be a significant contributor to ice/water reserves at the martian surface (Hecht et al., 2009), potentially poisoning and complicating In Situ Resource Utilization (ISRU) activities that would provide water for a crew.

Nearly the entire surface of Mars is covered in dust containing fine‐grained silicic particles (silicates), with martian dust, on average, being composed of 44.84 ± 0.52% SiO2 by weight (Berger et al., 2016; Montabone & Forget, 2017; Morris et al., 2006; Sim, 2017). Silica exists in both amorphous and crystalline forms (*i.e.*, a‐silica and c‐silica), with c‐silica generally considered more hazardous than a‐silica (Adamcakova & Mokra, 2021; Rabovsky, 1995). When small enough in diameter to reach the distal bronchioles and alveoli of the lungs, particles of c‐silica are considered biologically active. These particles are termed “respirable crystalline silica” (RCS) and have a diameter less than 5 μm (Hoy & Chambers, 2020; Li et al., 2022). Despite being considered the less hazardous form, a‐silica has also been found to induce an immune response, which mouse models demonstrated to be greatest in particles less than 1,000 nm in size (Kusaka et al., 2014; Pollard, 2016).

From Rocknest sand in Gale Crater, the combined amorphous components of the samples were found to represent a minimum abundance of 21%–22% by weight. In addition to being the largest component of these samples (42.88 wt %), SiO2 was found to have a disproportionately high percentage of the crystalline component when compared to the averages among the other components (Dehouck et al., 2014).

In contrast with the predominantly c‐silica found in martian dust, approximately 50% of Lunar dust is SiO2, but the bulk of it is in the vitreous (glassy) form, an especially toxic subtype of a‐silica (Agrell et al., 1970; Ghiazza et al., 2010). Both c‐silica and vitreous silica, unlike amorphous silica spheres, have irregular particles with sharp edges, stable surface radicals, and sustained release of hydroxyl radicals (Ghiazza et al., 2010). Studies investigating the health of commercial glass blowers, a population with high exposure to vitreous silica, have found an increased risk of developing silicosis and higher incidences of lung cancer (Sankila et al., 1990; Wani & Niyogi, 1968).

Silica has a high respirable size fraction, which results in a large percentage of inhaled particles penetrating into the alveolar region of the lung (Kerschmann, 2017; Weissman, 2022). Most studies regarding the health risks of silica inhalation investigate the c‐silica form (commonly referred to as quartz) because this is the most common form found on Earth and is a major occupational hazard (Baum & Arnold, 2023). Despite c‐silica's notoriety as a common cause of workplace‐associated pneumoconiosis, both c‐silica and a‐silica particles are phagocytosed by, and toxic to, macrophages, which leads to a cascade of inflammatory responses (Costantini et al., 2011; Pollard, 2016).

Silica inhalation has been found to have an association with adverse respiratory effects (*e.g.,* silicosis, chronic obstructive pulmonary disease), renal effects (silica nephropathy), immunological effects (numerous auto‐immune disorders), and an increased risk of lung cancer (RCS is classified as a Group 1 carcinogen) (Agency for Toxic Substances and Disease Registry, 2019; Department of Health and Human Services, 2002). Among these health effects, silicosis has the highest incidence. Silicosis is traditionally characterized by irreversible, progressive pulmonary fibrosis leading to restrictive lung disease (Baum & Arnold, 2023). Aside from serious surgical interventions (*e.g.,* lung transplant), there is no cure available for silicosis; thus, prevention is a necessity. Recent insights into the pathomechanisms are promising, but newly developed drugs have yet to be adopted as standard of care, and their efficacy remains uncertain (Adamcakova & Mokra, 2021).

On Earth, the most common form of silicosis is chronic simple silicosis, which occurs after over 10 years of moderate to low exposures to respirable crystalline silica (Pollard, 2016; Weissman, 2022). Chronic silicosis can be divided into two categories: simple silicosis and progressive massive fibrosis (Weissman, 2022). As silica exposure increases, the timeframe for the onset of silicosis symptoms becomes increasingly shorter. Accelerated (subacute) silicosis generally occurs after 5–10 years of high exposure, and acute silicosis (silicoproteinosis) can have an onset within weeks to a few years after exposure to extremely high concentrations of RCS (Pollard, 2016; Weissman, 2022). The acute form is the most severe form of silicosis (Pollard, 2016).

It has also been suggested that basaltic dust on the martian surface may cause even more deleterious health effects than that of pure silicas. Iron is a primary redox‐active metal on Earth, and likely on Mars, and both of its primary redox states can generate oxidative damage in the body. Ferrous iron (+2) is the most active of the Fenton metals, generating ROS in solution or causing direct biomolecule oxidation. Ferris iron (+3) is considered to be predominantly driven by direct lipid peroxidation (Cervini‐Silva et al., 2014; Horwell et al., 2015). Hurowitz et al. (2007) showed that basalt could produce hydrogen peroxide and also form hydroxyl radicals using ferrous iron (*i.e.*, the Fenton reaction). This occurred in two separate experiments: through immersion of mechanically pulverized basalt in water and via dehydroxylation of mineral surfaces in a dry environment. While significant amounts of reduced iron sulfides like pyrite have not been seen at Mars landing sites, it has been identified in numerous martian meteorites (Lorand et al., 2015; Tomkins et al., 2020). Pyrite is one of the most reactive minerals on Earth, generating high concentrations of ROS within water and simulated lung fluid, as well as a significant inflammatory stress response in A549 lung epithelial cells (Harrington, Hylton, & Schoonen, 2012, Harrington, Tsirka, & Schoonen, 2012). The ROS (*e.g*., hydroxyl radicals) produced are powerful oxidants that can induce oxidative stress and inflammation in the epithelial lining fluid of the lungs. This can then progress to causing lung disease in a manner similar to that of silica exposure previously described (Cohn et al., 2006; Hurowitz et al., 2007; Lelieveld et al., 2021). In fact, although the presence of silica is often indicated as the likely cause of “black lung” or coal worker's pneumoconiosis (CWP), the strongest correlation with disease formation and coal geochemistry, is with the presence of pyrite (Harrington et al., 2013; Huang et al., 2005). CWP is an incurable disease that has a predilection to forming pulmonary infiltrates, particularly in the upper lobes (Reichert & Bensadoun, 2009). CWP nodules develop as inflammatory and fibrotic reactions to inhaled dust (Weissman, 2022). Severe cases of CWP may result in progressive massive fibrosis (PMF) (Cool et al., 2023). As CWP progresses to PMF, smaller nodules may coalesce into larger masses of fibrotic tissue (Weissman, 2022). In the case of traditional CWP progression into PMF, important risk factors include high levels of cumulative respirable coal mine dust, pyrite, or respirable crystalline silica inhalation, the presence of small opacity disease, and a history of tuberculosis (Attfield et al., 2022).

Nanophase iron oxides, which similarly can cause tissue damage by the creation of ROS, were also found in martian soils by the Viking landers (Banin et al., 1993) as well as at most previous landing sites on Mars (*e.g.*, Rampe et al., 2016). Nanophase iron oxides are a significant component of the globally homogenous martian dust, and give Mars its red color (Ruff, 2022). A similar component of lunar dust caused noticeable health damage and technical challenges for the Apollo astronauts (Pohlen et al., 2022), and it could present similarly on Mars. It should also be noted that prolonged microgravity exposure on a mission to Mars has the potential to change astronauts' immune systems lending them more susceptible to disease (Crucian et al., 2018; Guéguinou et al., 2009) and also make pathological bacteria more virulent (Barrila et al., 2021; Singh et al., 2018; Zea et al., 2017). Since pathological bacteria are able to use excess iron to aid in their replication in a human host (Cassat & Skaar, 2013), the two mentioned considerations make increased iron exposure a concern for infection susceptibility for astronauts on long‐duration exploration missions.

Gypsum (CaSO_4_ · 2H_2_O) is a mineral that, on Earth, forms when sulfate‐ and calcium‐rich salt water evaporates (Szynkiewicz et al., 2010). Gypsum has been detected at several locations on Mars by the OMEGA instrument on ESA's Mars Express Orbiter, and its presence has been used as evidence that substantial volumes of liquid water were once present (Fishbaugh et al., 2007; Szynkiewicz et al., 2010). Initial gypsum findings were predominantly near Mars' North Pole, but recent findings of gypsum veins collected by Curiosity in a region known as Yellowknife Bay (just south of the equator) have bolstered evidence of widespread deposits (Nachon et al., 2014). Indeed, spectroscopic measurements from orbiters have detected hundreds of gypsum deposits globally, with some kilometers‐thick (*e.g.*, Carter et al., 2013). While gypsum (calcium sulfate dihydrate) is often a relatively innocuous substance, health risks increase when substantial quantities come into contact with mucous membranes (Roscina, 1971). Because gypsum is a hygroscopic compound (Zaragoza‐Benzal et al., 2023) that can harden quickly after absorbing moisture, short‐term exposure may cause mechanical irritation or GI blockage (Gosselin et al., 1984). If a large amount of gypsum has been ingested, it may be advisable to drink large amounts of water, glycerin, or gelatin solutions to delay “setting” and avoid obstruction (particularly of the pylorus) (Gosselin et al., 1984). Aside from major surgical interventions (*e.g.*, lung transplant or lobectomy), interventions for established disease are largely limited to symptomatic therapies (Sim, 2017). Long‐term exposure to, or exposure to high volumes of, inhaled gypsum can lead to an illness similar in pathophysiology to CWP (Sim, 2017). Other hydrated, hygroscopic sulfate minerals are also abundant on Mars such as kieserite, epsomite, and unidentified monohydrated sulfates (Cloutis et al., 2007; Vaniman et al., 2004). These have the potential to become hazardous to humans and irritating to the lungs, skin, and eyes (Giordani et al., 2022; Symbio, 2015). In potentially large quantities and in different forms than seen on Earth, these could present a novel health challenge.

Chromium, beryllium, arsenic, and cadmium are metals detected in martian soil that are hazardous to humans at low concentrations; however, their actual risk to humans at the concentrations detected by martian rovers is questionable (National Research Council, 2002). Nevertheless, they are included below for completeness to provide a thorough review and prepare for scenarios where higher concentrations of these metals are found.

Chronic inhalation of hexavalent chromium has been known to lead to a pneumoconiosis, resulting in cough, inflammation, fibrosis, and an increased risk of lung cancers such as squamous cell carcinoma (Sim, 2017). Chromium is naturally found in two forms, the hexavalent form [Cr(VI)] or the trivalent form [Cr(III)], the latter of which is more commonly found and not inherently toxic to cells. Chromium at an unknown valence state was found by Pathfinder on Mars, and was flagged as a potential toxin since the oxidation of Cr(III) to Cr(VI) is not implausible considering the highly oxidizing nature of martian soil and that Cr(VI) is highly toxic at low concentrations (National Research Council, 2002). Nevertheless, the abundance of Cr(VI) is debated by others, citing that it is highly unlikely to be found in martian soil based on Mössbauer spectrometer results from Gusev Crater and Meridiani Planum and the fact that Cr(VI) is unstable in the presence of Fe(II), which is abundant on Mars (Ming & Morris, 2017).

Whether exposure comes from pulmonary, circulatory, or dermal contact, Cr(VI) enters cells through anion transporters. In contrast, Cr(III) cannot transport in or out of cells. The presence of Cr(VI) intracellularly does not cause disease, but after cellular uptake, Cr(VI) is subsequently reduced by agents such as ascorbate (vitamin C), glutathione, or cysteine to Cr(III) (Sun et al., 2015). The reduction of Cr(VI) leads to the formation of hydroxyl radicals that cause oxidative stress and damage to the cell (Zhong et al., 2017), and since Cr(III) cannot be exported by the cell, accumulations of Cr(III) make Cr‐DNA adducts that can lead to chromosome breaks (Sharma et al., 2022).

In addition to pneumoconiosis, inhalation of Cr(VI) can cause acute diseases, such as allergic asthma and acute bronchitis, and it can also cause chronic diseases, such as nasal irritation and ulceration, emphysema, liver damage, and lung cancer. Cr(VI) toxicity may also occur through ocular, dermal, and oral routes which could result in hemorrhagic gastroenteritis, contact dermatitis, renal damage, neurological damage, corneal vesication and scarring, and congestion of the conjunctiva (Sharma et al., 2022; Sim, 2017; Sun et al., 2015; Wilbur et al., 2012; Zhong et al., 2017). No specific treatments are identified for Cr(VI) toxicity other than supportive measures and symptom management after exposure. Nevertheless, administration of vitamin C has been determined to be protective against ROS‐induced hepatotoxicity from Cr(VI) poisoning in mice (Zhong et al., 2017). Note that while vitamin C can be a pro‐oxidant, as is the case with the reduction of Cr(VI), Nowak et al. (2021) determined that at physiological concentrations, vitamin C primarily serves as an antioxidant in Fenton reactions, where it has the potential to be both pro‐oxidative and anti‐oxidative.

Beryllium content in martian dust also presents an area of potential risk as it can produce both acute and chronic chemical toxicity. Martian dust has been found to contain 10's of ppb of beryllium (National Research Council, 2002) ‐ approximately 18 μg/m^3^. Although the exact mechanism of the disease is unknown, inhaled beryllium is considered a Group 1 carcinogenic material (a human carcinogen) (Agency for Toxic Substances and Disease Registry, 2023).

Acute toxicity from beryllium inhalation occurs at 100 μg/m^3^ and is fatal in 10% of cases (Cummings et al., 2009). It presents with inflammation of the pulmonary system, leading to bronchiolitis, pulmonary edema, and pneumonitis (Stearney et al., 2023). Dermatologic contact can cause inflammation of the affected area, which causes irritation, ulceration, and subcutaneous granulomas (Stearney et al., 2023).

Chronic beryllium disease is hypothesized to be mediated by a Type IV hypersensitivity reaction wherein pre‐sensitized, beryllium‐specific CD4+ T cells form systemic granulomas indistinguishable from sarcoidosis. Although more research is needed in this area, it is known that individuals with a HLA‐DPB1‐Glu polymorphism show a higher level of sensitization, and toxicity can occur at continued exposure of levels as low as 0.2 μg/m^3^. Chronic beryllium toxicity presents similar to reactivated tuberculosis, with shortness of breath, unexplained coughing, fever, night sweats, and weight loss (Stearney et al., 2023).

Although no specific treatment currently exists for beryllium toxicity, measures can be taken to reduce the potential impacts (Balmes et al., 2014). Beryllium toxicity is generally a self‐contained disease, and removal of exposure within a timely manner after symptoms present can mitigate the potential risk of chronic disease. In addition, prednisone usage at 40 mg/day or every other day with reassessment has been shown to reduce inflammatory symptoms (Balmes et al., 2014). Methotrexate and azathioprine can also be used to reduce long‐term steroid use.

Arsenic is a semi‐metalloid found in martian soil, with analysis of meteorites showing concentrations ranging from 5 to 930 ppb (Lodders, 1998). Exposure to arsenic may have acute or chronic manifestations depending on the dose; the fatal human dose varies in case reports but is estimated to be 600 μg/kg/day (Agency for Toxic Substances and Disease Registry, 2007), with doses under 5 mg causing mild toxicity. Exposure routes include ingestion or inhalation of inorganic or organic arsenic. Arsine gas poses a particular danger due to the lethality of its inhalation at levels >10 ppm (Pakulska & Czerczak, 2006).

Ingested arsenic (either directly or by swallowing after mucociliary escalator clearance of contaminated inhaled particles) is well‐absorbed in the gastrointestinal tract (70%–90%) through passive diffusion. The primary target organ of arsenicals is the liver, where its reductive methylation is necessary for its elimination but generates reactive intermediates. These generate oxidative stress through the inhibition of complexes II and IV of the electron transport chain and are capable of methylating DNA, resulting in both short‐ and long‐term toxicity (Islam et al., 2015). Acute toxicity results in the sudden onset of abdominal pain and bloody or non‐bloody diarrhea followed by hypotension, similar to viral/bacterial gastroenteritis. Toxicity may progress to peripheral neuropathy, muscle cramping, seizure, and death in extreme cases (Ratnaike, 2003). Arsine gas, in particular, has been shown to result in hemolytic anemia and acute renal failure (Correia et al., 2009).

Symptoms of chronic arsenic exposure vary widely and are dose‐dependent, with manifestations usually presenting after 8–14 years. Skin lesions, including hyperpigmentation, arsenical keratosis, and leukonychia striata (*i.e.,* double white transverse lines in the nail beds) combined with peripheral neuropathy, are suggestive of chronic arsenic exposure. Sensory and/or motor peripheral neuropathy may progress to polyneuritis and motor paralysis of the distal extremities, and in extreme cases, to mental retardation and encephalopathy (Mehta, 2019). Arsenic is a Group 1 carcinogen at concentrations greater than 10 ppb. Trivalent arsenic compounds, in particular, may cause isolated and/or combined skin cancers (*e.g.,* squamous cell carcinoma, basal cell carcinoma, intraepidermal carcinoma), myelogenous leukemia, Hodgkin's disease, and internal cancers of the lung, liver, bladder, and kidney (Hall, 2002).

Treatment for severe acute arsenic toxicity includes gastric lavage and chelation therapy but is ineffective in chronic, progressed cases. Chelating agents used include dimercaprol, dimercaptosuccinic acid, and dimercaptopanesulfonic acid (Hall, 2002).

Cadmium is a transition metal found in martian soil, with meteorite measurements showing concentrations ranging from 2.1 to 95 ppb (Lodders, 1998). The half‐life of cadmium in the body is long, ranging from 10 to 30 years; therefore, it is considered a cumulative toxin with a tolerable weekly intake of 7 μg/kg/week. Exposure routes include ingestion and inhalation, with lethal oral doses ranging from 30 to 40 mg and lethal inhalation after 5 hr of inhaling 9 mg/m^3^ (Beton et al., 1966).

Cadmium's oral bioavailability ranges from 6% to 9%, while inhalation absorption ranges from 10% to 50%. Once absorbed, it may have deleterious effects on cell proliferation, differentiation, and apoptosis. It is capable of depleting reduced glutathione and binds sulfhydryl groups of proteins, including antioxidant enzymes, enhancing the production of reactive oxygen species. Its presence induces transcriptional changes in diverse cytotypes, including stress‐response genes and heat shock proteins (Luparello et al., 2010).

Acute high‐dose inhalation exposure of cadmium causes a self‐resolving flu‐like illness. More severe cases with higher doses of inhalation exposure can result in pulmonary edema and interstitial pneumonia, with death occurring in 20% of cases with pulmonary edema. Acute high‐dose oral exposure leads to a gastroenteritis‐like illness, with symptoms including diarrhea, nausea, and vomiting (Koons & Rajasurya, 2023).

Chronic cadmium toxicity has a wide range of dose‐dependent effects and may result from either route of exposure. Its deposition into the S1 segment of the proximal tubule of the kidney causes Fanconi syndrome; the resulting stress may cause tubulointerstitial nephritis, chronic nephritis, kidney stones, and renal cell carcinoma (Bernhoft, 2013). Vitamin D metabolism is also compromised, manifesting as osteomalacia and/or osteoporosis in a condition known as *itai‐itai* disease (Ogawa et al., 2004). Cardiovascular diseases, including hypertension and early atherosclerosis, as well as pulmonary diseases, including emphysema and various carcinomas, are possible (Bernhoft, 2013). Other effects include mild anemia, anosmia, and yellowing of the teeth (Liu et al., 1985). Chronic inhalation exposure, in particular, is associated with the development of chronic obstructive pulmonary disease.

Treatment for acute cadmium poisoning is limited. Gastric lavage and/or emesis are indicated if the exposure is responded to in a timely manner, but outside of these interventions, no treatment exists other than supportive care for acute toxic exposure. Chronic exposure has no treatment outside of care for adverse effects (Bernhoft, 2013).

## 3 Discussion

The small size of martian dust makes most dust particles more potent precipitators of human disease. Many of the identified toxins have a widespread distribution on the Red Planet, the effects of which need to be well studied prior to human occupation of Mars. Prevention and removal of dust exposure remains the most effective countermeasure. Dust mitigation technology development for lunar exploration may need modification to better suit the needs of martian missions. For example, Pohlen et al. (2022) discuss the use of multi‐stage HEPA air filters with magnetic filters to remove dust by attracting the nanophase iron. On Mars, filtration technologies also need to remove particulate oxidants and be effective during martian dust storms as they are crucial to supporting human life (*e.g.*, in spacesuit and habitat filters) and for in situ resource utilization fuel production systems (O’Hara, 2017). These systems must also be designed with limited resupply in mind, where the life cycle of critical components is sufficient for long duration missions. Effective dust mitigation will require a combination of strategies, including operational planning, passive controls, and active mitigation technologies (Johansen, 2020).

Secondarily, it is necessary to prevent disease from any mild chronic exposure, for which we propose iodine supplementation for perchlorate and vitamin C for Cr(VI) exposure (Table 1). Supplements and medications in astronauts need to be provided with caution, however, as the consequences of side effects are of a greater concern in this setting. For example, excess vitamin C can precipitate kidney stones, which are already at greater risk for astronauts living in the microgravity environment. It should also be noted that Mars is considered a restricted planet due to the possible existence of extant or extinct life, and while that opens the door for the potential existence of a pathogen (Cataldo et al., 2024), any discussion of the deleterious effects from an existing pathogen would be too speculative at this time.

For the presentation of acute disease, many large interventions, such as surgery, are not appropriate for space exploration in the immediate future. Even so, many treatments and medications may be used for the immediate therapy of multiple acute symptoms, such as bronchodilators for bronchospasms, gastric lavage and activated carbon for toxin ingestion, and rinses for dermal and ocular exposures. These treatments, in addition to other medications like prednisone, may also be indicated for other, non‐geological, medical issues that astronauts may face on a roundtrip mission to Mars.

It should be noted that while many of these hazards alone may be unlikely to cause significant disease, there is potential for combined exposures to amplify their deleterious effects. Chief of all is the effect on astronauts' lungs. The majority of these martian geological health hazards cause restrictive and fibrotic lung disease (*i.e.*, silica, basalt, gypsum, hexavalent chromium, and beryllium). Additionally, since astronauts are exposed to higher amounts of radiation leaving them susceptible to radiation‐induced pulmonary fibrosis (Christofidou‐Solomidou et al., 2015), the combined impact on astronauts' lungs could be more than just additive but synergistic. The development of any type of chronic pulmonary fibrosis on a long‐duration exploration mission would be detrimental to the mission and potentially fatal to astronauts.

## 4 Conclusions

The toxicity of lunar dust was an unpredicted health hazard during the Apollo missions. A mission to Mars does not have the luxury of rapid return to Earth for treatment, nor can it rely on flight surgeon ground support for care due to communication delays of up to 40 min round‐trip (Drake et al., 2010). Surface operations will require a far higher cadence of longer‐duration EVAs than previous missions to the Moon. These risks, together with both prolonged exposure to dust compared to lunar missions and the reduced ability to fight disease after long‐duration adaptations in microgravity and increased radiation exposure, make the hazard of dust a critical problem to solve for the successful and safe human exploration of Mars.

We highlight the chief recurring martian geological health hazards from literature and summarize their indicated risks, pathophysiologies, and treatments when encountered in hospitals on Earth. We emphasize limiting dust exposure as the primary, and most effective, means to prevent disease in astronauts. The potential health impacts from exposure to the martian surface must also be continuously considered as new planetary research is unveiled (*e.g.,* any results from the proposed Mars Sample Return mission). In order to adequately prepare for successful human exploration of the Red Planet, we must be ready to prevent and treat a host of medical issues that may arise while on the surface of Mars to mitigate risks and ensure both mission success and astronaut safety. To do that, we encourage scientists, engineers, and physicians from various disciplines to work together on a solution.

## Conflict of Interest

The authors declare no conflicts of interest relevant to this study.

## Data Availability

The data used to make Table 2 is available in Taylor (2013).
